# Prediction in reading: A review of predictability effects, their theoretical implications, and beyond

**DOI:** 10.3758/s13423-024-02588-z

**Published:** 2024-10-31

**Authors:** Roslyn Wong, Erik D. Reichle, Aaron Veldre

**Affiliations:** 1 School of Psychological Sciences, Macquarie University, Sydney, Australia; 2 Graduate School of Health, University of Technology Sydney, Sydney, Australia

**Keywords:** Brain imaging, Eye movements, Predictability effects, Prediction, Reading, Reading models

## Abstract

Historically, *prediction* during reading has been considered an inefficient and cognitively expensive processing mechanism given the inherently generative nature of language, which allows upcoming text to unfold in an infinite number of possible ways. This article provides an accessible and comprehensive review of the psycholinguistic research that, over the past 40 or so years, has investigated whether readers are capable of generating predictions during reading, typically via experiments on the effects of *predictability* (i.e., how well a word can be predicted from its prior context). Five theoretically important issues are addressed: What is the best measure of predictability? What is the functional relationship between predictability and processing difficulty? What stage(s) of processing does predictability affect? Are predictability effects ubiquitous? What processes do predictability effects actually reflect? Insights from computational models of reading about how predictability manifests itself to facilitate the reading of text are also discussed. This review concludes by arguing that effects of predictability can, to a certain extent, be taken as demonstrating evidence that prediction is an important but flexible component of real-time language comprehension, in line with broader predictive accounts of cognitive functioning. However, converging evidence, especially from concurrent eye-tracking and brain-imaging methods, is necessary to refine theories of prediction.

Reading is seemingly one of the simplest tasks that individuals engage in on a daily basis, yet understanding how it unfolds poses an ongoing challenge for psycholinguistic research. Successful reading is a complex process—comprehenders must decode written text into abstract representations of individual letters and words, retrieve their meanings from long-term memory, and integrate this information into the unfolding discourse representation. Despite its complexity, this entire process unfolds within hundreds of milliseconds with little conscious effort. One explanation for how readers are able to accomplish this feat so quickly and effortlessly lies in the idea of *prediction*.

In its broadest sense, prediction refers to any type of process that uses information about the past or present to estimate the immediately relevant future. In the context of language comprehension,^1^ readers encountering a phrase like “The day was breezy so the boy went outside to fly a . . .” can easily predict the word “kite” based on their prior knowledge and experiences (DeLong et al., 2005). These activated representations subsequently allow readers to process information faster when eventually encountered in the input stream. Psycholinguistic research over the past 40 or so years has provided evidence that readers rely on prediction—typically, by investigating the effects of *predictability*—that is, how well a word can be predicted from its prior context. This has led to several reviews in the literature, most of which focus on explaining the underlying mechanisms of prediction (Ferreira & Chantavarin, 2018; Huettig, 2015; Kuperberg & Jaeger, 2016; Pickering & Gambi, 2018; Ryskin & Nieuwland, 2023), or on reviewing empirical evidence from eye tracking (Staub, 2015) or electrophysiology (Van Petten & Luka, 2012). However, whether evidence of predictability effects means that prediction is a central component of real-time language comprehension remains under debate (Huettig & Mani, 2016). Many more studies of predictability effects across different areas of research have been published over the past decade, providing further important insights into the nuanced role of prediction during online processing. As such, the aim of the present review is to update the current state of knowledge about prediction and predictability effects, explore their theoretical implications, and outline potential future research directions. The overarching theoretical issues are whether effects of predictability can be taken to index genuine linguistic prediction during reading and whether, by extension, prediction must occur on some level for successful language comprehension.

This article is broadly organized into four sections. The remainder of this first section defines prediction in the context of both cognition and language comprehension. The following section reviews eye-tracking and brain-imaging research on the effects of predictability in studies of reading before exploring the mechanistic accounts of predictability effects provided by computational models of word identification and reading. The next section considers a number of prominent theoretical issues that provide more refined insights into the nature of predictability effects. The final section discusses the overarching theoretical issues mentioned above and outlines future research directions.

## Prediction in cognition

Prediction arguably plays a pervasive role in every aspect of human cognition. For example, in visual perception, individuals presented with a series of static images that imply motion, like a person diving off a cliff, anticipate ongoing mental representations of the unfolding event (Freyd, 1983; Senior et al., 2000). Similarly, in motor behavior, individuals predict upcoming actions based on internal forward models to facilitate motor control (Wolpert & Flanagan, 2001)—for instance, when using the fingertips to grip an object whose load is increased by a self-generated action such as a moving arm, individuals adjust their grip force without delay to prevent the object from slipping (Flanagan & Wing, 1997; Johansson & Cole, 1992). While early investigations of anticipatory mechanisms and representations began in the context of these lower-order sensory modalities of visual and motor cognition, prediction has since become implicated in more complex cognitive processes such as music processing (Land & Furneaux, 1997), emotion processing (Herwig et al., 2007), and theory of mind (Frith & Frith, 2006).

This experimental evidence is consistent with the view that the primary function of the brain is to anticipate future events and stimuli. While the classical view of the brain emphasized the role of lower-level sensory information in the construction of higher-level internal representations (Marr, 1982), the current twenty-first century perspective reverses this approach: “The brain is no longer viewed as a transformer of ambient sensations into cognition, but a generator of predictions and inferences that interprets experience according to subjective biases and statistical accounts of past encounters” (Mesulam, 2008, p. 368). Indeed, Clark (2013) posits that prediction offers a “deeply unified account of perception, cognition, and action” (p. 186) and goes as far as to surmise that the brain is fundamentally a “prediction machine.”

In line with this perspective, an increasing number of brain function models have become framed around predictive coding and minimizing prediction error (Clark, 2013; Friston, 2010; Hohwy, 2013, 2020). According to this framework, the brain is a hierarchically structured device that predicts sensory inputs via the interplay of backward (top-down) and forward (bottom-up) flows of information. The “backward” flow delivers predictions from higher to lower hierarchical levels based on what the system “knows” about the world and its current context such that incoming sensory signals that are consistent with these predictions are “explained away.” However, if there is a discrepancy between the sensory signals encountered and those predicted, the “forward” flow computes an error—that is, residual “unexpected” information that is propagated back up the hierarchy to refine top-down hypotheses about the current sensory data. Thus, the primary function of this multilevel bidirectional information exchange is to minimize overall prediction error or “free energy”—the probability of being in a state of surprise (Friston, 2010). This ensures that, in the long term, predictions about the world are being continuously updated and refined through knowledge and experiences (Bar, 2007; Lupyan & Clark, 2015), and an optimal model of the causes of incoming sensory signals are achieved across the different hierarchical levels (Friston, 2010).

Although empirical evidence that the brain implements predictive coding remains mixed (see Walsh et al., 2020, for a review), the general shift towards a predictive view of the brain is important for explaining cognitive processes for several reasons. Firstly, it has been posited that the brain would not be able to make sense of the world as rapidly as it does if it only relied on bottom-up information (Mesulam, 2008). Secondly, and perhaps more importantly, it has been argued that incoming sensory data is just too ambiguous and complex to deal with in a bottom-up fashion. As such, top-down processes, like the continual generation of predictions, help the brain perceive stability and coherence in its environment (Bar, 2007). Thus, prediction is now viewed as a, if not *the*, fundamental principle of human information processing.

## Prediction in language comprehension

Given that language processing is realized by the brain, it is perhaps unsurprising that prediction has been used to explain how real-time language comprehension unfolds so rapidly and effortlessly (Ferreira & Chantavarin, 2018; Huettig, 2015; Kuperberg & Jaeger, 2016; Lupyan & Clark, 2015; Pickering & Gambi, 2018). If the language processor is able to predict linguistic input based on prior knowledge and experiences, this information should facilitate subsequent processing when the input is eventually encountered. From this viewpoint, the role of prediction in theories of language comprehension should not be controversial. However, for decades, there have been doubts as to whether comprehenders can anticipate specific linguistic content in a way that goes beyond the effects of low-level intralexical priming.

The first major reason for this skepticism towards a central role for prediction in reading is that theories of language comprehension have traditionally espoused a strong bottom-up bias. For example, the classic modular views of language processing (Fodor, 1983; Forster, 1979) posit that words are recognized solely based on sensory input, and that context only has a postlexical impact, affecting the ease with which words are integrated into the unfolding sentence or discourse representation. Thus, the idea that contextual information could have a prelexical impact by encouraging the anticipation of upcoming linguistic input seemed untenable. Even more lenient views of language processing, such as those offered by the *cohort model* (Marslen-Wilson, 1987) and the *shortlist model* (Norris, 1994), which claim to be more interactive, still have a fundamentally bottom-up emphasis. In these models, contextual information can influence word recognition and lexical access, but only after the sensory input has activated an initial set of potential candidates.

The second major reason for why researchers have historically rejected the idea of prediction in reading lies in the inherently generative nature of language, which allows infinite possible linguistic expressions for each upcoming word of a sentence (Jackendoff, 2002; Morris, 2006). For example, consider the *cloze task*, in which individuals are instructed to continue a phrase or complete a sentence with the first word that comes to mind (Taylor, 1953). If most individuals converge on the same word, the context is considered *predictive* or *constraining* and the completion is deemed *predictable* or *high cloze probability*. However, most naturally occurring text is only weakly or moderately constraining (see, e.g., Provo Corpus; Luke & Christianson, 2016, 2018), so participants will often provide multiple plausible responses for these contexts (Bloom & Fischler, 1980). As such, predicting what will come next in a sentence is a relatively ineffective and potentially costly strategy unless contextual constraint is unusually strong—comprehenders are unlikely to make correct predictions very often and it may be more efficient to wait for the sentence to unfold naturally over the next few words (Forster, 1981; Jackendoff, 2002).

These antiprediction views, however, began to shift in the late 1990s. The modular views of language processing gave way to interactive views that emphasized the availability of contextual information even before the current sensory input had been processed (Ferreira & Lowder, 2016; McClelland, 1987; Morton, 1969; Sedivy et al., 1999). Meanwhile, the argument about generativity was challenged by the fact that, while few words are highly predictable in natural reading, many words or aspects of words are still moderately predictable. For example, consider again the first example presented in this article: “The day was breezy so the boy went outside to fly a kite.” Even though most comprehenders are unlikely to accurately predict in advance the word “fly” in this sentence, they can be fairly confident that a verb will be in this location. This syntactic information can then be used in conjunction with semantic and/or world knowledge to converge on a sufficiently plausible continuation. In line with these shifts in perspective, the past 40 or so years has seen growing experimental evidence of the predictive nature of the language processor.

The earliest evidence that comprehenders generate linguistic predictions during online processing comes from studies using lexical decision and speeded pronunciation (or “naming”) tasks. Many of these studies found that individuals were faster to make lexical decisions or name words that were contextually supported, such as “locker” in “John kept his gym clothes in his...,” compared with words that were not contextually supported, such as “closet” (Fischler & Bloom, 1985; Kleiman, 1980; Schwanenflugel & LaCount, 1988; Schwanenflugel & Shoben, 1985; Stanovich & West, 1983; Traxler & Foss, 2000). However, there are reasons to question the extent to which these findings from behavioral tasks can be generalized to normal reading given that the specific task demands of lexical decision and naming may recruit processing strategies that are not part of normal reading comprehension (Rayner & Liversedge, 2011).

Further evidence comes from studies using the *visual world paradigm* (Tanenhaus et al., 1995), in which participants’ eye movements are recorded as they look at a visual scene while listening to a sentence. In one of the most influential demonstrations, Altmann and Kamide (1999) presented participants with a visual context depicting a boy, a cake, and several other distractor objects while they listened to sentences such as “The boy will eat the cake” or “The boy will move the cake.” They found that, before the final word had even been presented, participants were faster to move their eyes towards the cake, the only edible object, in the “eat” compared with the “move” condition, suggesting that participants had predicted a compatible theme based on the selectional information conveyed by the verb. This finding of anticipatory eye movements based on contextual information has since been replicated in a number of visual world studies (Kamide et al., 2003; Kukona et al., 2011; see Huettig et al., 2011, for a review). However, questions have also been raised about whether these findings reflect genuine linguistic prediction given that the visual context places constraints on the potential candidates that can be heard in each sentence (DeLong, Troyer et al., 2014b; Huettig et al., 2011; Kutas et al., 2011). In other words, it is unclear whether evidence of prediction in visual world studies reflects anticipation due to linguistic or visual information.

Given the interpretational issues with these paradigms, it is unsurprising that researchers have turned to investigating lexical prediction during online processing via studies of reading, which have the potential to present a broad range of linguistic structures. This has coincided with the development of online methodologies such as eye-tracking and brain-imaging techniques. These methods are well-suited to the investigation of how real-time language comprehension unfolds because of their ability to provide continuous streams of data with high temporal resolution. In studies of reading, the clearest demonstration of prediction comes from evidence that a word has been activated even before it has been encountered. For example, DeLong et al. (2005) capitalized on a phonological rule in English where the indefinite article is realized as *a* before a consonant and *an* before a vowel, and presented sentences like “The day was breezy so the boy went outside to fly . . .” which were completed by either the predictable noun-phrase “a kite” or the less predictable, but plausible, noun-phrase “an airplane.” They found that neural activity in the form of an N400 component correlated with the predictability of the noun—the more predictable the noun, the smaller the neural activity, reflecting the ease of semantic processing. Critically, this inverse correlation was also obtained before the noun was presented—that is, on the preceding article that carried no semantic information. This led DeLong et al. to conclude that comprehenders had used the preceding context to anticipate the predictable noun, or at least its first phoneme, and by extension its appropriate preceding article (see also DeLong et al., 2012; Martin et al., 2013). While these findings could be taken as evidence of prediction, they should be interpreted with caution because subsequent studies failed to replicate these effects on the article (Ito et al., 2017; Nieuwland et al., 2018; but see Urbach et al., 2020). More generally, these manipulations are difficult to implement in English because there are few systematic linguistic rules that allow the properties of lexical items to influence preceding words (but see Fleur et al., 2020; Otten & Van Berkum, 2009; Van Berkum et al., 2005, for evidence from Dutch; Foucart et al., 2014; Wicha, Bates, et al., 2003a; Wicha, Moreno, et al., 2003b; Wicha et al., 2004, for evidence from Spanish).

Thus, most studies of reading in English have focused on demonstrating lexical prediction via the effects of *predictability—*how well a word can be predicted from its prior context. The basic idea is that, if a word can be predicted in advance of its presentation, then the way in which this word is processed when eventually encountered may depend on its level of predictability (Kutas et al., 2011). The most common approach to operationalizing predictability is via a word’s *cloze probability*, which can be determined by aggregating the responses provided for a given sentence context in a cloze task (i.e., the number of productions divided by the total number of responses; Taylor, 1953). Words with cloze values close to 1 are almost perfectly predictable in their contexts while words with low cloze values are less predictable. The next section presents a review of behavioral and neural research of the effects of word-level predictability during reading before exploring the mechanistic accounts of predictability effects provided by computational models of word identification and reading.

## Predictability effects in reading

### Evidence from eye-movement studies

Eye-movement recording is a noninvasive behavioral method for measuring online cognitive processing during reading. In the context of psycholinguistic research, eye movements offer certain advantages compared with traditional behavioral tasks. Firstly, participants do not need to complete a secondary task during reading, such as making decisions about words or naming them aloud, which could impose additional processing demands. Secondly, and perhaps more importantly, eye movements are fundamental to the reading process itself, meaning that ongoing lexical processing is captured by where and when readers move their eyes. It is therefore unsurprising that the eye-tracking methodology has been used extensively to investigate a range of language representations and processes (see Rayner, 1998, 2009, for reviews), including those related to predictability effects.

Ehrlich and Rayner (1981) first demonstrated the effects of predictability on eye movements in a pair of experiments that manipulated predictability in two ways. The first experiment presented the same target in two different contexts, while the second experiment presented two different target words in the same contexts, so that, in both manipulations, one target had a high cloze probability while the other had a low cloze probability. Across both experiments, Ehrlich and Rayner found that words that could be predicted from the preceding context were more likely to be skipped and to receive shorter reading times if fixated than words that could not be predicted, consistent with the idea that the predictability of a word determines the time required to process it.

Similar predictability effects have since been reported across a number of eye-movement studies using either experimentally controlled approaches, which involve manipulating a specific target word in a sentence context (see Staub, 2015, for a review), or corpus-based approaches, which involve analysing every word in a corpus of sentences or texts (Andrews et al., 2022; Luke & Christianson, 2016; see Kliegl et al., 2004, for evidence from German). Across these studies, predictability effects are typically observed on skipping (Abbott et al., 2015; Cutter et al., 2020; Fitzsimmons & Drieghe, 2013; Frisson et al., 2017; Rayner et al., 2001, 2011; Rayner & Well, 1996; Rich & Harris, 2023; Wong et al., 2022, 2024; see Brysbaert et al., 2005, for a meta-analysis) and first-pass reading measures such as first fixation and gaze duration (Fitzsimmons & Drieghe, 2013; Frisson et al., 2017; Rayner et al., 2001, 2011; Rayner & Well, 1996; Rich & Harris, 2023; Wong et al., 2022, 2024), suggesting that word predictability exerts its influence during early stages of processing (e.g., word recognition and lexical access; Vasishth et al., 2013). However, predictability effects are also observed on “late” reading measures such as total fixation duration (to the extent that it reflects second-pass time rather than first-pass time) and rate of regressions (Frisson et al., 2017; Rayner et al., 2011; Rayner & Well, 1996; Wong et al., 2022, 2024), possibly reflecting the influence of contextual information on later stages of processing (e.g., postlexical integration; Clifton et al., 2007). Thus, across different eye-movement studies, there is robust evidence that the predictability of a word is an important determinant of where readers look and for how long.

### Evidence from brain-imaging studies

Brain-imaging techniques allow inferences to be made about neural activity during online cognitive processing. Neural activity, which reflects communication between neurons, is an electrochemical process that generates electrical and magnetic activity, which can be detected via noninvasive electromagnetic techniques such as electroencephalography (EEG) and magnetoencephalography (MEG), respectively. Increases in neural activity are also accompanied by increases in blood flow to the relevant brain region, which can be detected via hemodynamic techniques such as positron emission tomography (PET) and functional magnetic resonance imaging (fMRI). These four techniques have been used widely in psycholinguistic research, with a significant number of contributions coming from EEG, especially when this technique is used to obtain* event-related potentials* (ERPs). Evidence of predictability effects during reading using these techniques are reviewed in turn, with a focus on ERPs.

#### Evidence from EEG

EEG is a noninvasive technique for measuring neural activity via electrodes placed on the scalp. It is well-suited to the study of language representations and processes because it provides high-resolution temporal information, which makes it possible to infer the stages of processing influenced by an experimental manipulation, even when participants’ only task is to read for comprehension. This technique can be used to obtain ERPs, which are small voltage fluctuations that are time-locked to an event or stimulus of interest, such as the onset of the presentation of a word, reflecting the summation of synchronized postsynaptic activity generated by a large population of neurons during information processing.

In EEG studies, predictability effects during reading have been shown to impact an ERP component known as the *N400*—a negative-going waveform with a centro-parietal distribution that begins 250 ms after the presentation of a word and peaks 400 ms after stimulus onset. Kutas and Hillyard (1980) initially discovered this neural waveform when they presented sentences like “He spread the warm bread with…” and found larger N400 amplitudes for semantically anomalous words like “socks” than acceptable control words like “butter.” Subsequent research revealed that the sensitivity of this ERP component was not restricted to semantic anomalies, per se: Larger N400 amplitudes were also observed for unpredictable compared with predictable words even when both were equally plausible. Kutas and Hillyard (1984) first demonstrated this when they presented sentences completed by either a high, medium, or low cloze probability target word and found an inverse relationship between predictability and the N400 component. That is, the higher a word’s cloze probability, the smaller the corresponding N400 amplitude, perhaps reflecting the fewer neural resources required to process a word. This graded relationship between predictability and the N400 component has been replicated in a number of studies (Brothers et al., 2015, 2017, 2020; Davenport & Coulson, 2011; DeLong, Quante, et al., 2014a; Federmeier et al., 2007; Hubbard et al., 2019; Kuperberg et al., 2020; Kutas, 1993; Lai et al., 2021, 2023; Rommers & Federmeier, 2018b; Thornhill & Van Petten, 2012).

The N400 component has also been shown to be sensitive to manipulations that go beyond predictability at the lexical level (see Federmeier, 2022; Kutas & Federmeier, 2007, 2011, for reviews). This ERP waveform is also modulated by predictability manipulations at the orthographic and phonological level (Delong et al., 2005; Ito et al., 2016; Laszlo & Federmeier, 2009; Martin et al., 2013), semantic level (Federmeier & Kutas, 1999; Kutas et al., 1984; Metusalem et al., 2012; Thornhill & Van Petten, 2012; Wlotko & Federmeier, 2015), and even for emojis (Weissman et al., 2024). The N400 is also sensitive to other lexical properties of words such as their frequency (Rugg, 1990), concreteness (Barber et al., 2013), and position in the sentence (Van Petten & Kutas, 1990, 1991). Indeed, the N400 component does not even appear to be language-specific because it is also elicited in response to any type of input that produces activity in long-term semantic memory, including faces and objects, numeric symbols, and environmental sounds (see Federmeier et al., 2016, for a review). Taken together, these findings suggest that the N400 component may be a default neural response to any potentially meaningful perceptual stimuli. Nonetheless, the fact remains that its amplitude is strongly correlated with a word’s cloze probability.

The timing of ERP predictability effects, however, suggests that they may occur relatively late in the time course of normal reading—that is, 400 ms after presentation of a word—or, at least, that they take some time to appear in the ERP record. Given that the typical eye fixation only lasts 200–250 ms, by 400 ms after a word has been fixated, readers have likely completed lexical access for that word and moved their eyes onto the subsequent word (Dimigen et al., 2011; Rayner & Clifton, 2009). As such, there remains an ongoing debate in the literature as to whether reduced N400 effects during reading reflect processing difficulty during the lexical or postlexical stage of processing (Kutas & Federmeier, 2011; Nieuwland et al., 2020). This, in turn, has implications for whether these effects can be taken as reflecting genuine prediction processes, which would be expected to influence early stages of processing. While there has been some evidence to suggest predictability effects on earlier ERP components, the reliability of these findings remains unclear (see Nieuwland, 2019, for a review).

#### Evidence from other brain-imaging techniques

Aside from EEG, other brain-imaging techniques used to measure neural activity during online cognitive processing include MEG, PET, and fMRI. However, evidence of predictability effects in studies of reading using these methods is comparatively scarce.

MEG is a noninvasive technique for measuring neural activity via magnetic sensors located a few centimeters from the scalp. The technique can be used to obtain *event-related fields* (ERFs), which, like their electrical counterparts (i.e., ERPs), provide high-resolution temporal information even during the simplest reading comprehension tasks. Additionally, because the anatomical origins of magnetic signals are easier to localize (see Van Petten & Luka, 2006, for a review), this technique also has the advantage of providing high-resolution spatial information. Predictability effects during reading have been shown to impact an ERF component known as the magnetic *N400* (N400m), which occurs around 300 to 500 ms poststimulus onset, corresponding to the N400 waveform observed in the ERP record. For example, Halgren et al. (2002) observed smaller N400m amplitudes for predictable compared with unpredictable words in a sentence reading task (see also Helenius et al., 1998, for evidence from Finnish), although it remains unclear whether this study separated lexical predictability from contextual plausibility. Similar to its electrical counterpart then, the N400m likely reflect a general response to meaningful perceptual stimuli (Ihara et al., 2007; Maess et al., 2006). More recently, predictability effects in the MEG record have also been linked to differences in oscillatory activity in left temporal regions of the brain (see Wang, Hagoort et al., 2018a, for evidence from Dutch). This region has also been shown to produce more similar patterns of neural activity for predictions of the same word in different contexts compared with predictions of different words (see Wang, Kuperberg et al., 2018b, for evidence from Chinese). However, these emerging areas of research and their links to predictive processing remain poorly understood.

The other techniques, PET and fMRI, measure changes in regional cerebral blood flow associated with brain activity. PET involves the injection of a radioactive, positron-emitting contrast agent and, perhaps due to this invasive procedure, is not commonly used in psycholinguistic research with no studies to date having investigated predictability effects during reading. fMRI, on the other hand, yields similar patterns of activation as the PET method (see Price, 2012, for a review), but is less invasive and does not involve radiation exposure. fMRI, also, has the benefit of providing high-resolution spatial information, although its low-resolution temporal information has limited its use in psycholinguistic research. In fMRI studies, predictability effects during reading have been linked to decreased activation in left frontal and temporal regions of the brain (Dien et al., 2008; see also Baumgaertner et al., 2002; Hartwigsen et al., 2017, for evidence from German).

Evidence from brain-imaging techniques provides important insights into the neural bases of predictability effects during reading. However, the extent to which these findings generalize to normal reading remains unclear. Brain-imaging studies often present stimuli using a *rapid serial visual presentation* (RSVP) *paradigm* in which sentences or texts are displayed one word at a time in the center of the screen at a fixed pace of 400–1,000 ms per word, which is assumed to provide sufficient time to separate neural responses to individual words. This presentation method clearly differs from normal reading in many aspects. Firstly, the word-by-word presentation format prevents readers from engaging in natural eye-movement behavior including skipping words, rereading text, and extracting upcoming information from the parafovea. Secondly, the fixed-pace presentation rate does not allow readers volitional control over the rate of input. Instead, words are typically presented for longer than the standard 200–250 ms duration of fixations, which could contribute to an increased likelihood of conscious prediction strategies (Dambacher et al., 2012; Wlotko & Federmeier, 2015). Finally, readers are required to maintain central fixation and suppress eye movements and blinks during the task. As such, reading under RSVP is quite unnatural and could impose additional processing demands that are not required in normal reading.

To partly address the limited ecological validity of RSVP, some brain-imaging studies have presented stimuli using a *self-paced reading* (SPR) *paradigm* in which sentences or texts are presented one word at a time but at the reader’s own pace (Ditman et al., 2007). Behavioral studies using this paradigm to investigate predictability effects have reported the classic processing benefits on reading times for expected compared with unexpected words, as measured by the latency of button presses (Brothers et al., 2017; Jongman et al., 2022). ERP studies using this paradigm have also revealed the expected smaller N400 amplitudes for predictable compared with unpredictable words (Ng et al., 2017; Payne & Federmeier, 2017b, 2019). Although these SPR findings appear to yield the same patterns of effects as the RSVP paradigm, they should also be interpreted with caution given the limitations of word-by-word presentation formats described above. Thus, it is possible that brain imaging studies using RSVP or SPR paradigms do not accurately capture the processes underlying normal reading, which is an important implication when considering discrepancies in the conclusions of eye-tracking and brain-imaging research.

### Evidence from concurrent eye-tracking and brain-imaging techniques

Researchers have begun to address the gap between brain-imaging studies and natural reading via co-registration methods, which involve the simultaneous recording of eye-movement and brain activity data from the same participants (see Himmelstoss et al., 2020, for a review). These methods of investigating continuous brain activity under natural reading have the advantage of enabling a more comprehensive assessment of the neural correlates underpinning online cognitive processing. They also allow for the analysis of brain activity that is time-locked to the onset of a fixation on a critical word, rather than to the onset of an externally triggered stimulus. However, only a small number of studies of reading have used co-registration methods to investigate predictability effects during reading. One approach has been the combination of eye movements and EEG to extract *fixation-related potentials* (FRPs), which has revealed the classic effects of cloze probability on both eye-movement measures and the amplitude of the N400 component (Burnsky et al., 2022; Kretzschmar et al., 2015; see also Dimigen et al., 2011; Kretzschmar et al., 2009, for evidence from German). Another approach has been the combination of eye movements and fMRIs to yield *fixation-related fMRIs* (Marsman et al., 2012), which has demonstrated that lexical predictability is both a significant predictor of reading times and involves a broad network of brain regions (Carter et al., 2019; see also Schuster et al., 2016, 2020, 2021; Weiss et al., 2023, for evidence from German). Although co-registration studies appear to replicate the findings of conventional brain-imaging studies, the interpretation of these findings should be qualified by the fact that events that occur at the same time in both records do not always necessarily reflect the same underlying processes. As with ERP data, the time-locked effects observed in FRP data, such as the N400, occur relatively late in the time course of normal reading. This means that not only have readers’ eyes likely moved onto the subsequent word, but the FRP waveforms risk being contaminated by neural processing from unrelated events. Nonetheless, with further ongoing development and refinement, the recording of neural activity during natural reading is likely to be a useful methodology for consolidating existing evidence of predictability effects in eye-tracking and brain-imaging research.

### Predictability effects within computational models of word identification and reading

On the basis of the above findings, a number of computer models have been put forward to formalize how predictability manifests itself to facilitate the reading of text. Computer models of reading can be grouped by the types of phenomena they attempt to explain—the identification of isolated words, the syntactic and semantic operations necessary to understand phrases and sentences, the processing required to represent more extended discourse, or how these processes are coordinated with vision and attention to produce the patterns of eye movements observed during reading (see Reichle, 2021, for a review). Because of this diversity and the fact that the models are formally implemented to varying degrees (e.g., flowcharts vs. computer programs), they differ considerably in terms of their assumptions about the processes involved in predictability effects. This section reviews computer models to provide a brief overview of their different assumptions and to highlight some of their theoretically interesting points of contrast. Firstly, models in each of the four previously mentioned groups developed to explain behavior during reading are considered, followed by models developed to simulate brain activity during reading.

#### Word-identification models

Models of word identification have largely been developed to explain the identification of single isolated words as measured using either lexical decision or naming tasks. As such, although the principles instantiated by these models are intended to “scale up" to explain how words are identified in natural reading, the details of how this occurs are unclear. Many central questions—such as how a word’s predictability might influence its identification—are either ignored or discussed at an abstract level. For example, the earliest word identification models were either silent about the role of predictability (Carr & Pollatsek, 1985; Coltheart, 1978; Glushko, 1979; Paap et al., 1982) or assumed that higher-level semantic and/or syntactic information could (somehow) influence lexical representations in a top-down manner (Forster, 1976; Morton, 1969). Although subsequent models were fully implemented and thus made more precise assumptions about how words are identified, the role of predictability in these models continued to be largely ignored (Gomez et al., 2008; Van Rijn & Anderson, 2003; Whitney, 2001). For example, the *interactive-activation model* (McClelland & Rumelhart, 1981) and most of its progeny (Coltheart et al., 2001; Davis, 2010; Perry et al., 2007; Zorzi et al., 1998) assume that words are represented by a hierarchical network of interconnected nodes, with information about the visual features of a printed word being propagated from feature nodes upwards through letter and word nodes in a highly interactive manner, with the word node receiving the most bottom-up activation eventually suppressing all other word nodes as it is identified. Complementing this description is the assumption that the activation of word nodes can also be supported through the top-down propagation of activation from higher-level nodes representing the context in which the words might be embedded. The latter assumption has not been implemented in models of word identification; however, as will be discussed below, it has been implemented in models of eye-movement control that incorporate variants of the interactive-activation model within their frameworks. One other interesting example of how word identification models might accommodate predictability effects is provided by the *Bayesian Reader* model (Norris, 2006). In this model, the frequency with which a given word has been previously encountered in text influences the prior odds of perceiving a given sequence of letters as that word. The linguistic context in which a word is embedded might similarly influence those prior odds, although precisely how this occurs also remains unspecified.

#### Sentence-processing models

The second category of reading models are those related to the processing of sentences. It is important to first note the starting assumption of these models which is that words have been accurately identified. The main objective of sentence processing models then is to explain how information about the meanings and syntactic categories of those words are used to construct the larger units of meaning corresponding to phrases and whole sentences. The earliest sentence processing models, for example, mainly used syntactic information from phrase structure rules to “decide” the likely syntactic categories of upcoming words in order to parse sentences as efficiently as possible (Frazier, 1978; Frazier & Fodor, 1978; Woods, 1970). By this conceptualization, syntactic information was privileged because it was assumed to influence sentence processing more rapidly than semantic information. Later models, however, challenged this assumption by positing that semantic and pragmatic information could be used rapidly to guide the parsing of sentences (Elman, 1990; Jurafsky, 1996; Just & Carpenter, 1992; Lewis & Vasishth, 2005; MacDonald et al., 1994; McClelland et al., 1989; Tabor et al., 1997; Van Dyke & Lewis, 2003). Although these models differ markedly in their implementation, they share the same underlying assumption that lexical, syntactic, semantic, and pragmatic information can be used jointly to constrain the identities of upcoming words.

One interesting variant of these sentence processing models that warrants discussion is *surprisal* (Hale, 2001; Levy, 2008), because it provides both an explanation for and a metric of the variation that is observed in word processing difficulty as a function of predictability. Unlike the accounts of sentence processing described above, which focus on the coordination of distinct cognitive operations that determine how words are recognized and integrated into unfolding sentence representations, surprisal theory is based on an ‘inferential” view of sentence processing according to which the primary role of the language processor is to assign a probability distribution over all possible continuations of a sentence (Shain, 2024). Processing effort is thus argued to be proportional to the magnitude of change in this probability distribution given by the amount of new information conveyed by each additional word. This process is also quantified by the metric of surprisal (a word’s negative log probability; see section Surprisal and entropy), which functions as a “*causal bottleneck* between representations and behavioral observables” (Levy, 2008, p. 1132). As with the other models of sentence processing, however, the assumptions of surprisal theory are general in two senses. The first is that a variety of factors (e.g., lexical, syntactic, semantic) that are distinct from the process of probabilistic inference likely contribute to language comprehension, with the precise mechanism(s) being underspecified (see Staub, 2024, for a review). The second is that predictions about the relative benefit or cost that are due to a word’s surprisal or predictability are often qualitative (e.g., a word will be identified more rapidly in Condition A than Condition B) or imprecise (e.g., words in Region A will be read more rapidly than words in Region B). It should be noted though that these two limitations reflect the fact that these models tend to emphasize the products of sentence processing rather than how the time course of sentence processing influences measures of online reading (e.g., eye movements).

#### Discourse-processing models

The limitations of sentence processing models are even more pronounced with models of discourse representation which often start with abstract, propositional representations of phrases or sentences and are even further removed from online processing. As such, these models generally have very little to say about the identification of specific words, but instead describe how the literal content of a text is used in conjunction with information in semantic memory to make a variety of inferences that are necessary for understanding and elaborating upon the text. For example, the earliest discourse representation models assumed that readers either use their knowledge of common story “scripts” to actively predict upcoming elements (Rumelhart, 1975) or rely upon the passive spreading of activation among related concepts in semantic memory to link the propositions of a text with whatever prior information might be relevant (Kintsch & van Dijk, 1978). Although recent models tend to emphasize the latter, specifying in more detail how spreading activation affords the making of inferences (Goldman & Varma, 1995; Kintsch, 1998; Myers & O’Brien, 1998; van den Broek et al., 1996), there are examples of models that maintain the core assumption that readers’ knowledge of causality plays an important role in understanding discourse (Fletcher & Bloom, 1988; Frank et al., 2003; Golden & Rumelhart, 1993; Langston & Trabasso, 1999). Like sentence processing models, however, all models of discourse representation are limited to making fairly coarse predictions about both the nature of the mechanism(s) that are responsible for predictability effects and how these effects manifest themselves during reading.

#### Models of the reading architecture

The final group of reading models are those that specify how word identification and, to a lesser degree, sentence processing interacts with the visual system and attention to determine when and where the eyes move during reading. Early models, which are not specific about these processes, make no attempt to explain predictability effects (Gough, 1972; Morrison, 1984; Reilly, 1993; see also McDonald et al., 2005). However, later models of the reading architecture, which provide more global descriptions of these processes albeit in an abstract, higher-level way, often make explicit provisions for explaining how predictability influences reading times. For example, the *E-Z Reader* model (Reichle et al., 1998, 2009, 2012) assumes that word *n* is identified in two successive stages, *L*_1_ and *L*_2_, with the mean time to complete the first stage, *t*(*L*_1_), being described by Eq. 1 and the mean time to complete the second stage, *t*(*L*_2_) being a fixed proportion of *t*(*L*_1_). As﻿ the top “branch” of Eq. 1 shows, word *n* can be “guessed” from its preceding sentence context, causing the value of *t*(*L*_1_) to equal 0 ms, with a probability equal to word *n*’s cloze probability (i.e., *predictability*_*n*_). Because most words have low predictability, however, the duration of *t*(*L*_1_) is most often a linear function of three free parameters (α_1_ = 124, α_2_ = 11.1, and α_3_ = 76), the logarithm of word *n*’s frequency of occurrence (i.e., *frequency*_*n*_), and its predictability.^2^ The model also assumes that, for word *n* to be predictable, all the preceding words must be identified and integrated; if the meaning of word *n* − 1 had not been integrated, the value of *predictability*_*n*_ is equal to 0. Thus, although highly predictable words (e.g., function words) can sometimes be guessed outright, the time required to identify most words is roughly an additive function of its frequency and predictability. Collectively, these assumptions allow E-Z Reader to accurately simulate the results of eye-tracking experiments (e.g., Rayner et al., 2004) which show that word frequency and predictability rapidly affect both the propensity to skip and the fixation durations on those words.

1 $$t\left({L}_{1}\right)=\left\{\begin{array}{l}0, \ \ \ \ \ \ \ \ \ \ \ \ \ \ \ \ \ \ \ \ \ \ \ \ \ \ \ \ \ \ \ \ \ \ \ \ \ \ \ \ \ \ \ \ \ \ \ \ \ \ \ \ \ \ \ \ \ \ \ \ \ \ \ \ \ \ \ \ \ \ \ \ \ \ \ \ \ \ \ \ \ \ \ \ with\ p={predictability}_{n}\\ {\alpha }_{1}-{\alpha }_{2}\text{ log}\ {frequency}_{n}-{\alpha }_{3}{predictability}_{n},\ \ \ \ \ \ \ \ \ \ \ with\ p=1- {predictability}_{n}\end{array}\right.$$

Other models of eye-movement control make similar assumptions about predictability effects. For example, the *SWIFT* model (Engbert et al., 2002, 2005) additionally assumes that how a word’s predictability affects its rate of processing depends upon whether lexical processing is in its first or second stage, and whether the word is being fixated or processed in the parafovea. This is accomplished using Eq. 2, where *F*_*n*_ is a variable that modulates the activation level of word *n* as a function of its current stage of processing and location. In Eq. 2, *t* is an index of time and *t*_*p*_ is the point in time when word *n* reaches its maximum activation, *f* (= 70.2) is a free parameter that increases the rate at which word *n* becomes active prior to this point, *θ* (= 0.11) is a free parameter that modulates the overall effect of predictability, and *k* is the point of fixation. Thus, in the top “branch” of Eq. 2, if word *n* is in the initial stage of processing and being processed from the parafovea, its rate of activation will be attenuated for more predictable words because such words require less bottom-up support to be identified. However, after word *n* reaches its maximum activation, its activation declines but more slowly for more predictable words. Together, these assumptions allow SWIFT to accurately simulate the kinds of predictability effects reported in eye-movement experiments (e.g., Kliegl et al., 2006).

2 $$F_n=\left\{\begin{array}{lc}+f\left(1-\theta\;{predictability}_n\right),\;\ \ \ \ \ if\;t < t_{p(n)}\ and\ k < n\\+f,\;\;\;\;\;\;\;\;\;\;\;\;\;\;\;\;\;\;\;\;\;\;\;\;\;\;\;\;\;\;\;\;\;\;\;\;\;\;\;\;\ if\ t < t_{p(n)}\ and\ k \geq n\\-\left(1+\theta\ {predictability}_n\right),\;\;\;\;\;\;\;if\;t\geq t_{p(n)}\end{array}\right.$$

One additional model of eye-movement control that warrants discussion is Li and Pollatsek’s (2020) *Chinese Reading Model* (CRM), which is designed to explain the patterns of eye movements observed during the reading of Chinese. Due to the unique writing system of the Chinese language, this model faces additional challenges that are not evident in the reading of languages that use alphabetic writing systems (see Reichle & Yu, 2024, for a discussion of these issues). Ignoring these script-related differences, however, the model is like both *Glenmore (*Reilly & Radach, 2006*) *and* OB1-Reader* (Snell et al., 2018) in that they combine variants of the interactive-activation model of word identification (McClelland & Rumelhart, 1981) described earlier and additional assumptions about the mechanisms that “decide” when and where to move the eyes. Importantly, the CRM also makes provisions for explaining predictability effects by adopting the assumption that, across time, *t*, the activation of a given word node *n*, *act*_*n*_(*t*), will increase at a rate that partially reflects its cloze probability. This assumption is described by Eq. 3, where *gain* (= 0.18) is a free parameter that determines the minimum effect of predictability. By adopting the preceding assumption, the CRM, like both E-Z Reader (Reichle et al., 1998, 2009, 2012) and SWIFT (Engbert et al., 2002, 2005), can provide quantitative accounts of eye-movement experiments (e.g., Rayner et al., 2005) that demonstrate how variation in the predictability of Chinese words influence various eye-movement measures on those words. Perhaps just as importantly, by doing this, the CRM makes good on the “promissory note” that, within the framework of the interactive-activation model (McClelland & Rumelhart, 1981), higher-level context can influence the time required to identify individual words.

3 $${act}_{n}\left(t+\Delta t\right)={act}_{n}\left(t\right)+{predictability}_{n}+gain$$

In considering the models of the reading architecture reviewed so far, it is necessary to acknowledge that Eqs. 1–3 describe three different ways in which a word’s predictability might influence its rate of processing and identification. Except for the CRM, which describes how words are identified in some detail, the other models provide only abstract descriptions of how predictability affects the rate of lexical processing. Perhaps for this reason, there have been two recent attempts to develop more comprehensive models of reading by embedding assumptions related to word identification, sentence processing, and/or discourse representation within the frameworks of models of eye-movement control. The first of these, *Über-Reader* (Reichle, 2021), incorporates an instance-based model of word identification (based on Ans et al., 1998) and a handful of assumptions about sentence processing and discourse representation within the framework of E-Z Reader (Reichle et al., 2012). Although this model does not currently explain predictability effects (see Reichle, 2021, pp. 505–506), two suggestions for addressing this limitation are offered. The first is that, in identifying a word, syntactic features consistent with the model’s predictions about the word’s syntactic category are used, in combination with the word’s orthographic features, to facilitate its identification. The second is that general semantic features related to the sentence or discourse topic might likewise be used in combination with the word’s orthographic features to facilitate its identification. These two approaches, if used concurrently, might allow for gradations in the specificity of the model’s predictions about the identities of upcoming words. The second of these more comprehensive models, *SEAM* (Rabe et al., 2023), embeds the sentence processing model of Lewis and Vasishth (2005) within the framework of the most recent version of SWIFT (Seelig et al., 2020). Although this model arguably provides a more sophisticated account of sentence processing than does Über-Reader, like the latter it also fails to provide an account of predictability effects, nor is there any suggestion for how SEAM might be modified to explain these effects.

#### Models of brain activity during reading

The computer models described so far have been developed to simulate behavior during reading and, as such, cannot be directly applied to the explanation of brain activity during reading. In recent years, progress has been made towards the development of models that are capable of simulating the patterns of neural data observed during reading in brain-imaging studies and the role of predictability in this process. Perhaps unsurprisingly though, these models have predominantly been informed by neural activity measured using ERPs given the extensive research conducted using the EEG method compared with other brain-imaging techniques. Computational models of language electrophysiology can be ascribed to two general categories—large-scale models that are based on natural language corpora and small-scale models that are based on specific theoretical considerations.

Large-scale computational models link ERP activity to complex neural networks that have been trained to predict the next word in large corpora of spoken and/or written texts (Devlin et al., 2019; Radford et al., 2019). For example, a number of studies that have derived surprisal estimates of predictability from language models have shown that these probabilistic measures predict N400 amplitudes during natural reading (Aurnhammer & Frank, 2019; Frank et al., 2015; Michaelov et al., 2021). However, only one study by Lindborg and Rabovsky (2021) has suggested that this is because changes in the internal activation states of these models are predictive of N400 amplitudes. Overall, because surprisal, an output measure of these models, is typically used to predict neural activity only, it remains unclear mechanistically how a word’s predictability is related to brain activity during reading.

Small-scale computational models, on the other hand, link ERP activity to changes within internal hidden layers of neural networks (see Nour Eddine et al., 2022, for a review). Early models, which were based on the processing of single words and word pairs, focused on simulating the N400 observed in response to lexical factors and simple priming manipulations (Cheyette & Plaut, 2017; Laszlo & Armstrong, 2014; Laszlo & Plaut, 2012; Rabovsky & McRae, 2014). However, because the process of word identification in these models is assumed to involve the mapping of orthographic features onto distributed semantic representations, the details of how sentence-level variables such as predictability influence this ERP component are largely ignored. For example, one class of models posits that N400 amplitudes reflect the amount of semantic activation produced by bottom-up input (Cheyette & Plaut, 2017; Laszlo & Armstrong, 2014; Laszlo & Plaut, 2012), while another class posits that they reflect the discrepancy between the internal semantic state of the model and the semantic features of the input (Rabovsky & McRae, 2014). Later models, which were based on the processing of sentences, focused on simulating the N400 observed in response to broader contextual factors including predictability (Brouwer et al., 2017, 2021; Fitz & Chang, 2019; Rabovsky, 2020; Rabovsky et al., 2018). According to these models, N400 amplitudes reflect either the amount of change in an internal neural network representation layer (Brouwer et al., 2017, 2021; Rabovsky, 2020; Rabovsky et al., 2018) or the difference between the next-word prediction of the model and the input actually presented (Fitz & Chang, 2019). But even though some of these models are able to simulate the effects of predictability on the N400, the magnitude of change associated with this component is generally computed outside the model’s architecture, so the direct link between predictability and brain activity during reading in these models remains unclear.

In summary, computer models of reading have the capacity to provide insights into the patterns of behavioral and neural data during reading in eye-movement and brain-imaging studies. However, most models to date lack sufficient quantitative detail to explain how predictability manifests itself to facilitate the reading of text. More specifically, existing models of brain activity have largely been informed by ERP data, so it remains unclear how their assumptions would apply to data from other brain-imaging techniques. Thus, the ongoing development and refinement of computer models of reading are important for evaluating the feasibility of different accounts of predictability effects and for motivating new empirical research. It will also be a critical challenge for future models to simulate the effects of lexical and contextual factors on both behavioral and neural data with minimal additional assumptions.

## Theoretical issues relating to predictability effects

Eye-tracking and brain-imaging research, together with computer models of reading, provide robust evidence that the predictability of a word has a strong influence during online processing. This section explores several prominent theoretical issues that provide more refined insights into the nature of predictability effects during reading.

### What is the best measure of predictability?

One important theoretical question that has implications for understanding the nature of predictability effects is how to best measure subjective predictability as experienced by human comprehenders. This review so far has described studies of reading that operationalize a word’s predictability through the measure of cloze probability, which is calculated by aggregating responses provided for a given sentence context in a cloze task (Taylor, 1953). This section evaluates the cloze probability metric in more detail and reviews several alternative computational approaches including transitional probability and the information-theoretic metrics of surprisal and entropy.

#### Cloze probability

Cloze probability is the favored metric for estimating a word’s predictability by virtue of the fact that it is derived from human comprehenders themselves, thereby capturing their expectations of what word will come next in a given sentence context. This means that, unlike more objective approaches, which are influenced by the co-occurrence of words in text, cloze probability provides a subjective but purer measure of what researchers are interested in: how predictable a word is in a given context. There are, however, a few limitations that have been identified about this metric. Firstly, cloze probability can be noisy and subject to response biases—for instance, failure to follow task instructions or motivational issues may lead some participants to provide overly simple responses and others to produce overly elaborate or unnatural responses. Secondly, it is an open question whether cloze probability reflects a genuine measure of comprehenders’ online predictions. Because the cloze task is an offline, untimed procedure, participants are able to engage in conscious reflection and, consequently, strategic task completion compared with online reading during which typical fixations last 200–250 ms. Thirdly, and relatedly, it remains unclear what exactly the cloze task captures. Staub et al. (2015) found that participants generated cloze responses faster when contextual constraint was higher, suggesting that the task captured a race between lexical units to accrue contextual activation rather than the effects of predictability per se. Luke and Christianson (2016) also found that the lexical, positional, and semantic properties of a word influenced participants’ cloze responses (see also Smith & Levy, 2013), reflecting the general cognitive constraints of a production-based task, although they noted that the majority of the variance was explained by cloze values over and above these individual predictors. Thus, although cloze probability is arguably the most widely used measure of word predictability, perhaps because of the limitations outlined, some researchers have turned to more objective computational approaches.

#### Transitional probability

One alternative measure that has been suggested as a potential determinant of a word’s predictability is forward *transitional probability *(TP), the conditional probability that word *n* will occur given word *n* − 1. Unlike cloze probability, this low-level statistical information is derived from text corpora and is independent of high-level contextual information. For example, based on a typical corpus of texts, the verb *accept* is followed by the noun *defeat* more often than the noun *losses*. TP effects were first demonstrated by McDonald and Shillcock (2003a), who observed that high TP nouns yielded shorter first fixation durations than low TP nouns, although this benefit did not extend to gaze duration or skipping (see also McDonald & Shillcock, 2003b, for similar evidence using corpus-based analyses). However, a subsequent study by Frisson et al. (2005) suggested that these apparent effects of TP may actually be effects of cloze probability because, although the nouns presented in McDonald and Shillcock’s (2003a) study were very low in cloze probability, high TP nouns were rated as 10 times more predictable than low TP nouns (.08 vs .008). When Frisson et al. tested this possibility by presenting McDonald and Shillcock’s verb–noun phrases in neutrally or highly constraining contexts, they found cloze probability effects on the early measures of first and single fixation duration, in addition to a TP effect on gaze duration. Notably, however, cloze probability varied substantially between the high and low TP nouns in both context conditions, implying that this TP effect was present only when cloze probability had not been controlled. A subsequent experiment in which cloze probability was controlled revealed significant effects of cloze probability but not TP. On the basis of these findings then, word predictability appears to be more accurately captured by cloze probability rather than TP (but see Andrews & Reynolds, 2013; Li et al., 2021).

#### Surprisal and entropy

Another set of measures that has been used to estimate a word’s predictability is *surprisal* and *entropy*, which are derived from statistical (e.g., *n*-gram, phrase structure grammars, neural networks) or large language models (e.g., masked, autoregressive) trained on large corpora with the objective of predicting the upcoming word of a sequence given prior context. This approach is gaining prominence in the literature because it allows probability estimates to be generated for items across the entire distribution, including those at the lower end that might otherwise be associated with a cloze probability of zero. The first measure, *surprisal,* captures the extent to which a word is unexpected in its context (Hale, 2001; Levy, 2008) and is measured by taking the negative log probability of a word given its prior context: *surprisal* (*w*_*i*_) = *−log p*(*w*_*i*_|*w*_*1*_*…w*_*i−1*_). Words with higher surprisal, a lower probability of occurring in a sentence, are harder to process, while words with lower surprisal, a higher probability of occurring in a sentence, are easier to process. Consistent with this account, increasing word surprisal has been linked to longer fixation durations (Amenta et al., 2023; Cevoli et al., 2022; Lowder et al., 2018; Onnis et al., 2022; Smith & Levy, 2013) and larger N400 amplitudes (Aurnhammer & Frank, 2019; Frank et al., 2015; Michaelov et al., 2021). The second measure, *entropy,* captures the degree of uncertainty about how a sentence will unfold (Shannon, 1948) and is measured by taking the negative sum of the probabilities of all outcomes in a sentence, *p*(*x*), multiplied by the logarithm of the probabilities of the outcomes: *entropy* $H\left(X\right)=-\sum\nolimits_{x\in X}\;p\;(x)\;\log_{2\;}p\;(x)$. Entropy is higher when all possible continuations are probable and lower when there is certainty about the upcoming continuation. Because entropy captures the degree of certainty before a target word is reached, it has also been used to calculate a related metric, *entropy reduction*, (*H*_*i*_ − *H*_*i-1*_), which provides an index of the amount of information gained with the identification of each additional word (Hale, 2003, 2006, 2016). Currently, however, behavioral and neural evidence of a relationship between processing effort and either entropy (Aurnhammer & Frank, 2019; Cevoli et al., 2022; Roark et al., 2009; van Schijndel & Linzen, 2018) or entropy reduction (Frank, 2013; Frank et al., 2015; Linzen & Jaeger, 2016; Lowder et al., 2018; Wu et al., 2010) remains somewhat mixed.

#### Future directions

Researchers have access to a number of methodological options for measuring a word’s predictability in context; however, determining which measure best captures subjective predictability as experienced by human comprehenders remains an ongoing debate. The emergence of language models over the past decade, in particular, has unlocked a new set of tools for explaining processing effort during reading. In light of the increasing use of computational approaches to examine predictability effects, it is important to highlight some specific issues that may require further empirical investigation.

Firstly, in comparison to surprisal, which is the most commonly used computational metric for estimating word predictability, substantially less research has focused on the potential role of entropy and, by extension, entropy reduction. The disproportionate focus is somewhat surprising given that entropy captures the state of the model’s predictions *before* a target word is reached, arguably providing a better index of predictability than surprisal, which captures the state of the model’s prediction *when* a target word is reached (Aurnhammer & Frank, 2019; Cevoli et al., 2022; Karimi et al., 2024; Pickering & Gambi, 2018). Given that overall evidence of the effect of entropy on processing effort is mixed, however, it would be useful for future research to clarify the link between this metric and different processing effort indices, and whether it makes a contribution to word processing that is distinct from that of surprisal.

Secondly, it remains unclear whether computational metrics based on language models explain variance in processing effort to the same extent as cloze probability derived from human comprehenders. Studies that have explicitly considered this issue have yielded mixed findings: Computational estimates appear to perform worse (Frisson et al., 2005; Smith & Levy, 2011), equivalently (Chandra et al., 2023), or even better (de Varda et al., 2023; Hofmann et al., 2022; Michaelov et al., 2021) than human estimates of predictability. These discrepant outcomes, however, could reflect methodological differences between the studies, including the language model used, the corpus on which the model was trained, the type of analyses conducted, and the processing indices being investigated. Thus, it will be important for future research to consider these technical choices more systematically to assess the predictive power of computational metrics of predictability.

Finally, to the extent that language models can yield reliable estimates of subjective predictability, it also remains an open debate which models, if any, are analogous to the human comprehension system. Recent findings suggest that statistical language models perform more poorly than large language models in accounting for behavioral (de Varda et al., 2023; Shain et al., 2024) and neural indices of processing effort (Merkx & Frank, 2021; Michaelov et al., 2021), even though, intuitively, the former seem to be more cognitively plausible because of their incremental processing of words and constraints on working memory which mirror that of human comprehenders (Keller, 2010; Merkx & Frank, 2021). Even among large language models, smaller models with fewer parameters appear to explain reading behavior better (de Varda et al., 2023; Shain et al., 2024), suggesting that overly powerful models with unlimited access to large sequences of words may overestimate contextual influences on predictability effects compared with human comprehenders (Oh & Schuler, 2023). With the ongoing development and refinement of large language models, it will be important for future research to consider which computational approaches reliably approximate subjective predictability while also being neurocognitively plausible models of language comprehension.

### What is the functional relationship between predictability and processing difficulty?

Given the link between predictability and how efficiently a given word is processed, another theoretical question that has been the focus of substantial research is how to quantitatively define this relationship. The simplest account is that the relationship between predictability and processing effort is linear. That is, if predictions about upcoming words are generated according to a probability matching scale, increases in predictability should correspond to decreases in processing effort. An alternative account is that the functional relationship is logarithmic, meaning that the ratio of predictability, rather than its raw difference, determines the processing effort between items—a difference in predictability of .05 compared with .1 should yield the same effect on processing as a predictability difference of .5 compared with 1. The key difference between these two accounts then is how they expect contextual information to affect the processing of very unpredictable words—while logarithmic accounts expect even small differences in low predictability to lead to processing effects, linear accounts expect these effects to be negligible because processing effects are driven primarily by highly predictable words. Determining which function underlies predictability effects is important for understanding whether readers use context to predict upcoming words in proportion to their probability or to preactivate information across the entire lexicon.

Even though linear and logarithmic views of predictability make different predictions about how word processing unfolds, the empirical record on this question is currently mixed. Evidence of a linear predictability relationship comes from Brothers and Kuperberg’s (2021) meta-analysis of eye-movement data (*N* = 218), which found that linear models of predictability as estimated by cloze probability provided a better fit to the data than logarithmic models. This was confirmed in two further tasks using SPR and cross-modal picture naming, although these tasks clearly differ from normal reading in many aspects. Other studies using computational estimates report evidence of a logarithmic predictability relationship (Shain et al., 2024; Smith & Levy, 2013; Wilcox et al., 2023; see also Rayner & Well, 1996, for evidence using cloze probability). For example, Smith and Levy (2013) found a logarithmic effect of predictability across six orders of magnitude (i.e., 1 to .000001) when using a tri-gram co-occurrence measure to analyze eye-tracking and self-paced reading measures from naturalistic corpora. Finally, it has been suggested from EEG data that predictability effects could even follow an additive linear and logarithmic function. This superimposed function was first reported by Szewczyk and Federmeier (2022), who observed that the logarithmic function captured variability in the N400 waveform for unexpected words, while the linear function captured additional attenuation in the component for expected words.

Thus, there is no clear consensus on the nature of the functional relationship between predictability and processing effort, although most findings appear to favor a logarithmic account. These mixed outcomes could reflect the impact of two key factors that have differed across investigations of these issues. Firstly, studies have differed in their operationalization of word predictability, with most studies supporting a logarithmic account using computational metrics. As previously mentioned, this approach provides estimates for items across the entire probability distribution, including for words that are unlikely to be produced by human comprehenders, but which are critical to the differentiation of linear and logarithmic effects. Secondly, studies have varied in their use of either constructed or naturalistic language materials. Most studies favoring a logarithmic view have utilized the latter, which, unlike constructed language materials, allow for word-by-word modelling and are therefore able to cover the critical low end of the probability distribution that distinguishes these competing accounts. It should be noted though that naturalistic texts are constrained by their lack of experimental control, allowing words to vary on multiple uncontrolled dimensions that may influence the predictor of interest (Angele et al., 2015; Rayner et al., 2007). As such, the discrepant findings observed across the literature could reflect the methodological choices made by researchers, which appear to be more conducive to producing logarithmic predictability effects.

#### Future directions

It remains to be determined whether predictability effects are driven primarily by words at the upper or lower end of the probability distribution. Future investigations of this question should focus on clarifying the extent to which the methodological choices made by researchers contribute to the linking function that relates predictability and processing effort. For example, the reanalysis of existing data using different predictability estimates has the potential to provide valuable insights. Indeed, when Shain (2024) reanalyzed Brothers and Kuperberg’s (2021) SPR data using surprisal estimates derived from the large language model GPT-2, they found that the data were consistent with logarithmic over linear predictability effects. More generally, although most studies have focused on measuring processing effort behaviorally as a function of predictability, few studies have examined this issue via neural indices of processing effort. While it is possible that different processing indices capture distinct underlying functions (e.g., Burnsky et al., 2022; Federmeier, 2022; Kretzschmar et al., 2015), additional insights from neural methods could potentially inform this debate of whether predictability effects are linear or logarithmic in nature.

### What stage(s) of processing does predictability affect?

Another theoretical question that has interested researchers relates to the stage(s) of processing that is/are affected by effects of predictability. One way in which researchers have explored this issue has been by investigating the effects of predictability on word recognition in sentence contexts. In general, word identification in context can be conceptualized as comprising three stages: (1) the *prelexical* *processing stage*, which involves any processing that occurs before lexical access, such as the encoding of a word’s visual and orthographic features; (2) the *lexical processing* or *lexical access stage*, which involves matching this abstract information to a representation in the mental lexicon; and (3) the *postlexical* *processing stage*, which involves any processing that occurs after lexical access. Several factors that have been identified as having an impact on distinct stages of word identification are word frequency, parafoveal preview, and stimulus quality. Investigating how these factors interact with contextual information has the potential to provide insights into the stage(s) of processing affected by word predictability. Based on the additive factors logic proposed by Sternberg (1969), interactive effects would suggest that these variables affect the same stage of processing, while additive effects would suggest that these variables affect different stages of processing (but see McClelland, 1979). This section reviews evidence from studies that have investigated how these factors interact with predictability, with a focus on data from eye-movement and ERP methods.

#### Predictability and word frequency effects

The frequency of a word, as indexed by how often it occurs in corpora of spoken and/or written texts, is one of the most robust predictors of the speed and accuracy of word identification. High frequency words yield more skips (Drieghe et al., 2005; Rayner & Raney, 1996) and receive shorter first fixation and gaze durations (Inhoff & Rayner, 1986; Rayner & Duffy, 1986) than low frequency words, reflecting the early influence of word frequency on lexical processing. High frequency words also yield smaller N400 components than low frequency words, especially when words are presented in lists (Barber et al., 2004; Grainger et al., 2012; Rugg, 1990) or at the beginning of a sentence (Dambacher et al., 2006; Van Petten & Kutas, 1990). While it is debated whether this ERP component necessarily reflects lexical processing (Kutas & Federmeier, 2011; Nieuwland et al., 2020), frequency effects have also been reported in the earliest N1 time range, a negative-going waveform that occurs approximately 130–200 ms poststimulus onset (Hauk & Pulvermüller, 2004; Sereno et al., 1998). Thus, across different methodologies, evidence of frequency effects has been taken as evidence of lexical access (Sereno & Rayner, 2000, 2003).

Given the robust early influence of word frequency, researchers have gained insights into the temporal locus of predictability effects by investigating whether predictability interacts with frequency effects. Under modular views of language processing (Fodor, 1983; Forster, 1979), which imply that predictability affects a different stage of processing (i.e., postlexical) compared with frequency, these two variables should show additive effects on word recognition based on Sternberg’s (1969) additive factors logic. Under interactive views of language processing (McClelland, 1987; Morton, 1969), however, which posit that predictability and frequency affect the same stage of processing, there should be evidence of an early interaction between these two variables. That is, as formalized by the inferential view of probabilistic models (Hale, 2001; Levy, 2008; Norris, 2006), which propose that frequency effects function as predictability effects when context is absent, the predictability effect should be more pronounced for low compared with high frequency words. Computational models of reading also vary in the assumptions they make about the relationship between frequency and predictability. For example, according to the E-Z Reader model discussed earlier, a word’s predictability and frequency modulate the rate of lexical processing in an additive manner (see Eq. 1). However, the relationship between word predictability and frequency is less clear in both SWIFT (Eq. 2) and the CRM (Eq. 3) because, in both models, the rate of lexical processing itself is affected by other variables (e.g., concurrent word processing in SWIFT and inhibition from orthographically similar words in the CRM).

The question of whether word frequency interacts with contextual information during reading has been examined empirically in three ways. The first method involves reaction time measures, which were mainly used in early behavioral studies. For example, Stanovich and West (1981, 1983) used a naming task to test the effects of predictability on pronunciation latencies of high and low frequency words. They found significant main effects of predictability and frequency, as well as an interaction in the direction described above—that is, larger predictability effects for low compared with high frequency words—suggesting that contextual information did interact with word frequency to determine how easily a word was identified. Similar patterns of interaction effects were also reported in studies using lexical decision tasks (Becker, 1979; West & Stanovich, 1982). However, several factors limit the generalizability of these behavioral studies including the presentation method and task-specific demands, which may recruit different strategic processes compared with normal reading.

The next two methods involve studies of eye movements and ERPs grouped by whether they use categorical or continuous manipulations of these variables. Studies that use categorical manipulations rely on constructed language materials that factorially manipulate cloze probability and corpus frequency. Eye-movement studies using this controlled design have reported main effects of each variable on fixation durations but no significant interaction (Ashby et al., 2005; Hand et al., 2010, 2012; Rayner et al., 2001, 2004; Staub, 2020; Staub & Goddard, 2019; but see Sereno et al., 2018). In contrast, ERP studies, have reported interactive effects of both variables on the N400 component, with larger effects of predictability observed for low compared with high frequency words (Dambacher et al., 2006; Sereno et al., 2020; Van Petten & Kutas, 1990; see also Sereno et al., 2020, for evidence on the P1). Thus, there appear to be two distinct patterns of combined predictability-frequency effects on online processing measures, which have implications for determining the temporal locus of predictability. The additive effects observed in the eye-movement record indicate that word predictability may not operate at the same “lexical access” stage of processing as word frequency; however, the opposite is suggested by the interactive effects observed in the ERP record. The findings of Kretzschmar et al.’s (2015) FRP study may provide some insight into this discrepancy: While there were additive effects of both variables on first fixation and gaze duration, there was a predictability effect on the N400 component, but no frequency effect or interaction. The distinct overall processing patterns may therefore reflect different underlying functions of eye movements and ERPs (see also Burnsky et al., 2022; Federmeier, 2022).

Studies that use continuous manipulations of predictability and frequency, on the other hand, rely on naturalistic language materials, which allow researchers to analyze each word of a sentence or text rather than a single critical region. Early studies which calculated cloze probability for each word found only additive effects of predictability and frequency (Kennedy et al., 2013; Whitford & Titone, 2014). More recent studies using computational metrics based on language models have reported similar findings (Goodkind & Bicknell, 2021; Shain, 2024; but see Shain, 2019). For example, Shain (2024) found that predictability as indexed by surprisal derived from GPT-2 was a significant predictor of reading times from three different modalities (eye movements, SPR, and MAZE^3^) from six naturalistic corpora, independently of unigram (log-frequency) probability.

In summary, the nature of the relationship between predictability and frequency, as well as the specific stage(s) of processing affected by predictability effects, remain far from clear. The discrepant findings observed across the literature could again reflect the methodological differences including the predictability estimates, language materials, and processing indices being investigated. The following sections discuss other factors that might provide clearer insights into the stage(s) of processing affected by word predictability.

#### Predictability and parafoveal preview effects

This section has focused on the effects of predictability on word identification that are derived from text that includes, or is prior to, the fixated word. However, readers also extract information from upcoming words in the parafovea.

Parafoveal processing is typically investigated in eye-movement studies using the gaze-contingent *boundary paradigm* in which a target word is replaced by a preview word in its location until the reader’s eyes cross an invisible, predefined boundary at the end of the pretarget word (Rayner, 1975). Because readers are not usually aware of this display change due to saccadic suppression (Matin, 1974), the paradigm can be used to infer the types of information extracted parafoveally by comparing reading measures on target words between conditions displaying different types of information in parafoveal vision. Boundary paradigm studies have revealed a characteristic *parafoveal* *preview effect*, whereby targets following a valid preview (i.e., identical) compared with invalid preview (e.g., an unrelated word, pseudoword, or nonword) receive facilitated processing—although part of this effect also reflects a preview cost from encountering misleading information parafoveally (Kliegl et al., 2013).^4^ Numerous experiments have converged on the finding that readers are able to preprocess orthographic, phonological, morphological, and some semantic information from upcoming words (see Andrews & Veldre, 2019; Schotter et al., 2012; Vasilev & Angele, 2017, for reviews). Given that parafoveal preview effects are generally interpreted as emerging during prelexical and lexical processing, investigating whether contextual information interacts with parafoveal information can provide insights into the stage(s) of processing affected by word predictability.

Balota et al. (1985) conducted the first eye-movement study to investigate this question. Participants read sentences such as “Since the wedding was today, the baker rushed the wedding cake/pie”, which were completed by either a predictable or unpredictable but plausible word that was preceded by one of five parafoveal previews: valid (i.e., the target word itself, e.g., “cake”); a nonword that was visually similar to this target (e.g., “cahc”); the other possible target word (e.g., “pies”); a nonword that was visually similar to this other target (e.g., “picz”); or an unrelated and visually dissimilar word (e.g., “bomb”). Balota et al. found higher skipping rates for predictable compared with unpredictable words only when following a valid identical preview and, to a lesser extent, an invalid but visually similar preview, suggesting that predictability-based skipping required a preview that at least visually matched the predictable word (but see Drieghe et al., 2005). They also found that the predictability benefit on gaze duration was present only following a preview that was identical or visually similar. This interactive pattern, whereby the predictability effect is eliminated for invalid but not valid previews, has been observed on early reading measures across a number of studies (e.g., Juhasz et al., 2008; Luke, 2018; Staub & Goddard, 2019; Veldre & Andrews, 2018b; White et al., 2005b) and has been taken to suggest that predictability effects depend on the ambiguity of perceptual input during early orthographic processing (Staub & Goddard, 2019; but see Parker et al., 2017; Parker & Slattery, 2019). When readers are denied valid previews, orthographic processing of the target word must unfold entirely in foveal vision, meaning that perceptual information is too clear for predictability information to have an effect on early processing stages. In contrast, when readers have access to valid previews, orthographic processing of the target word begins before they are directly fixated, based on degraded visual input with the support of contextual information. On the basis of these eye-movement studies, word predictability appears to exert its influence during the earliest parafoveal stages of processing.

The relationship between predictability and parafoveal preview has been investigated to a lesser extent in ERP studies because the RSVP paradigm does not allow for the processing of upcoming words in the parafovea. The *visual hemi-field flanker RSVP paradigm* partly addresses the limitations of RSVP by presenting each word of a sentence centrally, flanked to the left and right by the preceding and following word, respectively (Barber et al., 2011). Studies using this methodology have reported graded N400 predictability effects for words presented in parafoveal vision which are subsequently attenuated when the same words appear foveally (Payne et al., 2019; Stites et al., 2017). Another more ecologically valid approach has been the co-registration of EEG and eye movements under natural reading conditions. Using this methodology, Burnsky et al. (2022) found the expected interaction on early reading measures, that is, predictability effects for valid but not invalid previews, while the same interaction on the N400 was observed for fixations time-locked to the onset of the pretarget, but not target, word. This neural evidence provides further support for the idea that word predictability may exert its effects when words are viewed in parafoveal vision (Staub & Goddard, 2019).

In summary, there appears to be reliable evidence across methodologies that predictability affects the processing of parafoveal information, suggesting that the temporal locus of word predictability is either during prelexical processing or very early lexical processing. If this is the case, predictability would also be expected to interact with factors that affect the earliest stages of letter or word processing, such as stimulus quality. This possibility is considered in the next section.

#### Predictability and stimulus quality effects

The quality of a stimulus has been shown to influence how long readers spend processing a word (e.g., contrast reduction, Becker & Killion, 1977; dot-pattern degradation, Meyer et al., 1975). One naturalistic way to manipulate stimulus quality is *contrast reduction*, which involves adjusting the contrast between a word and its background. A number of studies have found that words presented in faint text tend to receive longer first fixation and gaze duration than words presented normally (Drieghe, 2008; Reingold & Rayner, 2006; White & Staub, 2012), suggesting that stimulus quality affects early stages of word identification, during which visual features are encoded and abstract letters are computed (Besner & Roberts, 2003). If it is the case that word predictability modulates an early stage of processing, as implied by its interaction with parafoveal information, predictability should also interact with the quality of the visual stimulus.

The question of whether stimulus quality interacts with higher-order information including predictability has received relatively limited investigation. The earliest relevant findings come from studies using lexical decision which reported that priming effects were larger for degraded compared with intact targets (Balota et al., 2008; Borowsky & Besner, 1993). On the assumption that a semantic prime in a lexical decision task functions like contextual information during normal reading in that both involve the preactivation of general semantic information and/or a specific lexical unit (Staub, 2015), these findings suggest that word predictability may operate at the same stage of processing as stimulus quality. Recent studies investigating this issue using a more typical predictability manipulation have revealed a different set of findings. Staub (2020) recorded participants’ eye movements as they read sentences containing target words that were factorially manipulated for predictability and stimulus quality. While they found main effects of both variables, the interaction effect was very small and statistically unreliable, albeit in the expected direction—that is, the predictability effect was larger for faint compared with normal text. A subsequent FRP study conducted by Burnsky et al. (2022) using a similar design replicated these eye-movement effects. In the FRP record, they found an effect of predictability, but no evidence of a stimulus quality effect or an interaction between the two variables. Thus, in the two studies to directly investigate this issue, there appears to be little evidence of the interactive effects that would be expected if predictability affects the same stage of processing as stimulus quality.

#### Future directions

These findings across three separate literatures demonstrate that it remains unclear which precise stage, or stages, of processing is/are affected by a word’s predictability. While it appears that word predictability does not influence lexical processing given that it does not reliably interact with frequency effects, evidence that it influences an earlier stage of processing is mixed given that it interacts with preview validity but not stimulus quality. A tentative conclusion is that predictability effects operate at a very specific early stage of processing when words are viewed in parafoveal vision, which would be consistent with the idea that readers engage in the prediction of upcoming words in advance of their presentation. Further research into the relationship between predictability and other variables that have been linked to early stages of word or letter processing may provide additional insights (e.g., font difficulty, Rayner et al., 2006; Staub, 2020; letter rotation, Blythe et al., 2019; text mirroring, Chandra et al., 2020).

One final approach to addressing this theoretical question could be through the investigation of the distributional effects of predictability and other factors on parameters of the ex-Gaussian distribution. The ex-Gaussian distribution is a convolution of a normal distribution and an exponential distribution specified by three parameters: *μ* (the mean of the distribution), *σ* (the variability of the distribution), and *τ* (the degree of the rightward skew of the distribution). When fit to individual data, this distribution indicates whether an experimental manipulation impacts processing effort by shifting the distribution and/or changing the skew. Word predictability has been found to impact the *μ* parameter only in fixation duration distributions (Sheridan & Reingold, 2012; Staub, 2011; Staub & Benatar, 2013) with high predictability words shifting the distribution to the left. Word frequency (Reingold et al., 2012; Staub et al., 2010) and parafoveal preview (White & Staub, 2012) have been found to impact both the *μ* and *τ* parameters, while stimulus quality manipulated via contrast reduction has been found to impact the *μ* parameter (White & Staub, 2012). Although these distributional effects appear to suggest that predictability is more similar to stimulus quality than to frequency or parafoveal preview, these findings should be interpreted with caution, as these effects have not been assessed within a single study (see, e.g., Shain, 2024). Further research into this approach thus has the potential to inform how early effects of predictability arise during reading.

### Are predictability effects ubiquitous?

Another ongoing question in the psycholinguistic literature is whether predictability effects are an automatic consequence of online processing. In order to address this issue, it is necessary to investigate whether and how predictability effects interact with factors such as interindividual differences among readers, age, and task demands.

#### Predictability effects and individual differences

One factor that has been shown to mediate predictability effects during reading is individual differences among readers (Huettig, 2015). In particular, these processes have been linked to language expertise, with a number of studies demonstrating larger predictability effects and/or more efficient use of predictability information in highly- compared with less-skilled readers, as indexed by measures of literacy (Ng et al., 2017; Steen-Baker et al., 2017) and verbal ability (Cheimariou et al., 2021). Relatedly, studies of non-L1 English adults have revealed smaller predictability effects in L2 compared with L1 speakers (see Ito & Pickering, 2021, for a review). Yet there is concurrent evidence to suggest that, even among L1 speakers, poorer readers, as indexed by their reading speed, show more pronounced predictability effects than better readers (Ashby et al., 2005; Slattery & Yates, 2018; but see Payne & Federmeier, 2019). Poorer readers’ impoverished lexical representations (Perfetti, 2007; Perfetti & Hart, 2002) may require them to rely more on contextual information for word identification (Perfetti & Lesgold, 1979; Stanovich, 1984). More generally, predictability effects have been linked to cognitive resources such as cognitive control (Zirnstein et al., 2018) and attention (Brothers et al., 2017; Hubbard & Federmeier, 2021) among other factors that have typically been investigated using the visual world paradigm (e.g., working memory, Huettig & Janse, 2016; Ito et al., 2018). Thus, the magnitude of predictability effects during online processing appears to vary among readers, and is systematically influenced by individual differences.

#### Predictability effects and age

Predictability effects during reading have also been shown to interact with age, a factor that lies at the intersection between language skills and cognitive abilities. On the one hand, normal aging is associated with the accumulation of language skills (e.g., vocabulary, Alwin & McCammon, 2001; Verhaeghen, 2003; word-related knowledge, Salthouse, 1993), which implies that older adults should be more sensitive to contextual information compared with younger adults (Payne et al., 2012; Ryskin et al., 2020). On the other hand, however, aging is accompanied by an array of changes in visual (e.g., reduced central and peripheral acuity, general visual pathology; see Owsley, 2011, for a review), and cognitive processes (e.g., lower processing, reduced attention and executive control, and smaller working memory capacity; see Verhaeghen, 2013, for a review), which suggest that older readers should use predictability information less efficiently compared with their younger counterparts. Perhaps because of these competing trajectories, investigations of how aging impacts predictability effects to date have yielded mixed findings.

Eye-movement studies suggest that older readers show equivalent, if not stronger, predictability effects during reading compared with younger readers (Andrews et al., 2022; Cheimariou et al., 2021; Choi et al., 2017; Rayner et al., 2006; Veldre et al., 2022; see Zhang et al., 2022, for a review). This has been argued to reflect an overall *risky reading strategy* according to which older adults rely on contextual information during reading to compensate for declines in visual and cognitive abilities (McGowan & Reichle, 2018; Paterson et al., 2020; Rayner et al., 2006; but see Choi et al., 2017; Veldre et al., 2021, 2022; Zhang et al., 2022). ERP studies, on the other hand, report the opposite pattern of effects. Compared with younger readers, older adults consistently show either smaller N400 effects, delayed N400 effects, or both (Dave, Brothers, Swaab et al., 2018a, Dave, Brothers, Traxler et al., 2018b; Federmeier & Kutas, 2005; Payne & Federmeier, 2017a; Wlotko et al., 2012; Wlotko & Federmeier, 2012, see Payne & Silcox, 2019; Wlotko et al., 2010, for reviews), suggesting that they are in fact less sensitive to contextual information. With age, there is also limited evidence of the late frontal positivity, which has been taken to index the processing cost of making an incorrect prediction (Dave, Brothers, & Swaab, 2018a; Wlotko et al., 2012), although a subset of older adults with higher verbal ability appear to show evidence of this neural waveform (Dave, Brothers, Traxler et al., 2018b). While these mixed findings may reflect differences in the stimuli presentation rate and format across methodologies, it appears that, at least in samples of older readers, predictability effects may not always be observable during online processing.

#### Predictability effects and task demands

Another important factor that has been shown to modulate predictability effects during reading is task demands, which have been investigated using several different approaches. One approach has been to use instructions to explicitly manipulate readers’ engagement with prediction during reading, for example, by asking participants to read sentences in a passive comprehension block and then in an active prediction block where they are instructed to predict passage-final words and report the accuracy of their predictions. Using this paradigm, a number of studies have found enhanced N400 predictability effects, reflecting greater facilitation for expected input when predictive strategies were encouraged in active compared with passive blocks (Brothers et al., 2017; Dave, Brothers, Traxler, et al., 2018b; Lai et al., 2023). It is unclear, however, whether these graded effects extend to the late frontal positivity, which indexes the cost of an incorrect prediction (see Lai et al., 2023, for a discussion). A second approach used by researchers has been to vary information in readers’ broader linguistic environment to implicitly modulate their propensity to use prediction during reading. Using this type of manipulation, Brothers et al. (2017) observed diminished effects of predictability on self-paced reading times when the broader linguistic environment contained a low proportion of confirmed predictions (<20%), suggesting that readers do not always rely on predictive strategies when this strategy is likely to be futile (Kuperberg & Jaeger, 2016). While these studies investigating predictability effects under different task instructions have typically used less natural reading paradigms, they do provide further support for the notion that predictability effects during online processing may be more complex and multifaceted than previously assumed.

#### Future directions

Evidence of predictability effects is near ubiquitous during online processing; however, it is likely that these effects also vary across groups and individuals, and under different circumstances. It will be important for future research to investigate whether there are other factors that could potentially mediate predictability effects during reading. For example, language production mechanisms have been implicated in predictive processes during language comprehension (Dell & Chang, 2013; Federmeier, 2007; Pickering & Garrod, 2007), but most studies investigating this link have been in the context of the visual world paradigm and/or correlational in nature (see Huettig, 2015; Pickering & Gambi, 2018, for reviews). It will also be informative for future research to assess whether a language’s writing system has an impact on predictability effects. Most research to date has been conducted using alphabetic languages. For instance, consider Chinese, a logographic script written as strings of characters with no spacing cues to demarcate word boundaries, which raises questions about whether and how word predictability information might be used during online processing (e.g., Rayner et al., 2005). Investigations of these issues will provide further insights into whether predictability effects unfold automatically during reading given certain factors and task demands.

### What processes do predictability effects actually reflect?

In this article so far, evidence of predictability effects has been taken to index genuine prediction processes. This section reviews this claim in further detail before discussing some alternative processes that may account for predictability effects during reading: graded preactivation, integration, and probabilistic inference. Determining what processes underlie predictability effects is tied to the broader debate about the cognitive architecture of the human language comprehension system.

#### Lexical prediction processes

The most intuitive account of predictability effects is that they reflect prediction processes—if a word can be predicted in advance of its presentation, then the way in which this word is processed when eventually encountered may reflect its level of predictability (Kutas et al., 2011). Most psycholinguistic research to date has explicitly, if not implicitly, defined prediction as the “all-or-none process of activating a linguistic term (a word) in advance of perceptual input” (DeLong, Troyer et al., 2014b, p. 632). This predictive process, termed *lexical prediction*, is assumed to involve the prediction of a specific lexical candidate, which is accompanied by processing benefits if readers’ predictions turn out to be correct but processing costs if they turn out to be incorrect. Most evidence of predictability benefits outlined so far in this article could be taken as support for the first half of this assertion, even if most naturally occurring text is neither predictive nor constraining (Luke & Christianson, 2016). However, support for the second half of this assertion—the processing costs when unpredictable words violate a more expected completion in their context—remains more elusive. Eye-movement studies have found minimal evidence of prediction error costs across various studies using constructed (Frisson et al., 2017; Wong et al., 2022; but see Wong et al., 2024) and naturalistic language materials (Andrews et al., 2022; Luke & Christianson, 2016; but see Cevoli et al., 2022). In contrast, ERP studies have reported evidence of enhanced neural activity for prediction violations in the form of a positivity, approximately 600–900 ms poststimulus, with an anterior scalp distribution (Brothers et al., 2017, 2020; Delong et al., 2011; DeLong, Quante, et al., 2014a; Federmeier et al., 2007; Hubbard & Federmeier, 2023; Hubbard et al., 2019; Kuperberg et al., 2020; Kutas, 1993; Lai et al., 2021, 2023; Rommers & Federmeier, 2018a; Thornhill & Van Petten, 2012; see Van Petten & Luka, 2012, for a review). This neural component has been distinguished from a positivity with a parietal scalp distribution that occurs in the same time frame for unpredictable words that are anomalous in strongly constraining contexts (Brothers et al., 2020; DeLong, Quante, et al., 2014a; Kuperberg et al., 2020; Thornhill & Van Petten, 2012; see Van Petten & Luka, 2012, for a review). Although the late frontal positivity appears relatively late in the time course of normal reading, one hypothesis is that it reflects higher-order processes related to the suppression or inhibition of the incorrectly predicted word (Federmeier et al., 2007; Kutas, 1993; Ness & Meltzer-Asscher, 2018). However, because there is also evidence of this ERP waveform for unexpected input in weakly to moderately constraining contexts where the most expected completion is unlikely to have been preactivated (Brothers et al., 2015; Davenport & Coulson, 2011; Hubbard et al., 2019; Thornhill & Van Petten, 2012), another hypothesis is that it also reflects integration of the unexpected input and/or updating of the broader discourse representation (Brothers et al., 2015; DeLong, Quante, et al., 2014a; Kuperberg et al., 2020). While this discrepancy in observing prediction error costs could in part be attributed to the differing methodologies and processing indices, the absence of conclusive evidence of the processing costs that would be expected to accompany incorrect predictions remains a challenge to the argument that readers make use of lexical prediction during reading.

#### Graded preactivation processes

Another account that has emerged in more recent years is that predictability effects do not involve specific lexical predictions but rather the partial preactivation of upcoming words (Brothers & Kuperberg, 2021; Federmeier, 2022; Luke & Christianson, 2016; Staub, 2015; Staub et al., 2015). This predictive process, termed *graded prediction,* is assumed to involve the passive activation of information related to the current discourse as part of the natural organization of long-term memory in response to incoming linguistic input (Federmeier, 2022). As such, multiple lexical candidates can be generated for each upcoming word of a sentence without incurring any processing costs if they turn out to be incorrect. This type of prediction is accounted for by the evidence of predictability benefits considered so far in this article, as well as several other findings. Firstly, a number of studies have shown that readers preactivate relevant information about upcoming words, including at the syntactic level (Cutter et al., 2022; Dikker et al., 2010; Lau et al., 2006; Staub & Clifton, 2006; see Ferreira & Qiu, 2021, for a review) and orthographic and phonological levels (Delong et al., 2005; Ito et al., 2016; Laszlo & Federmeier, 2009; Luke & Christianson, 2012; Martin et al., 2013). Secondly, as described above, few studies have found processing costs for incorrect predictions and have instead observed facilitated processing for unpredictable words when semantically related to the best completion (Andrews et al., 2022; Federmeier & Kutas, 1999; Frisson et al., 2017; Luke & Christianson, 2016; Thornhill & Van Petten, 2012; Wlotko & Federmeier, 2015; Wong et al., 2022, 2024). Thus, there is growing evidence to support the notion that readers engage in the graded prediction of upcoming words, even if their full lexical identity cannot be predicted from prior context.

Although graded prediction differs in its assumptions about how language processing should unfold compared with lexical prediction, it is unlikely these two accounts reflect two completely distinct processes. An emerging proposal is that prediction is a dynamic process involving the activation of multiple sources of information over different time courses (Burnsky et al., 2022; Federmeier, 2022; Huettig, 2015; Pickering & Gambi, 2018; Szewczyk & Federmeier, 2022). For example, Federmeier (2022) posits two processing modes for language comprehension: *connecting*, which involves linking incoming linguistic input with long-term semantic memory to activate information in a graded and parallel manner, and *considering*, which involves using comprehension strategies like prediction to transform initial graded semantic representations into more stable multidimensional representations. Pickering and Gambi (2018) similarly propose a two-systems account of prediction: *prediction-by-association*, which is characterized by spreading activation between related concepts (Collins & Loftus, 1975; Neely, 1977), and *prediction-by-production*, which involves using the production system to covertly anticipate upcoming linguistic content (see also Dell & Chang, 2013; Huettig, 2015; Pickering & Garrod, 2004, 2013). Thus, under these dual-systems accounts of prediction, the initial stage of (graded) preactivation is passive but obligatory, which prepares the language processor for the subsequent active but optional stage of (lexical) prediction. This second stage is nonobligatory because it can depend on readers having sufficient time (e.g., slower presentation rates, Ito et al., 2016; Wlotko & Federmeier, 2015) and resources (e.g., language skills, cognitive abilities, and age). In other words, predictive processes occur along a continuum: At any given point in a sentence, readers may either preactivate general semantic information or, with the availability of time and resources, activate a more specific word form.

#### Postlexical integration processes

Another account of predictability effects is that they reflect *postlexical* *integration processes*, that is, when a comprehender combines linguistic information that is activated as a result of processing the current input with a representation of the preceding input (Kutas et al., 2011; Pickering & Gambi, 2018; Van Petten & Luka, 2012). Consider again the first example presented in this article: “The day was breezy so the boy went outside to fly a . . .” for which the high cloze completion “kite” is processed most efficiently. According to the prediction view, “‘kite” receives facilitated processing because comprehenders are more likely to have activated it in advance of its presentation, making it easier to process than a low cloze completion like “airplane.” According to the integration view, however, “kite” is still easier to process even if it has not been predicted because comprehenders are more likely to have activated linguistic information pertaining to “kite” based on the prior context, but importantly, not the lexical item itself. As such, when “kite” is encountered, it is easier to integrate by combining the word’s meaning with the representation of the preceding input.

While these accounts of predictability effects can be distinguished theoretically, it is difficult to find empirical evidence that is compatible with prediction but not integration processes. As discussed in the section Prediction in language comprehension, there are a handful of EEG studies that demonstrate clear evidence of prediction by revealing that a word has been activated even before it has been encountered by the comprehender (DeLong et al., 2005, 2012; Martin et al., 2013). However, these studies are in the minority due to the difficulty of designing such manipulations in English, and because some of these effects have failed to replicate. As such, evidence of facilitated processing for predictable words across eye-tracking and brain-imaging methodologies could be compatible with an integration account given that they involve measuring online processes that occur at, and not before, the critical target. More generally, the fact that predictability affects “early” eye-movement measures such as skipping and first fixation duration does not provide unequivocal evidence for an early locus of predictability effects because a substantial amount of lexical processing can occur when the word is in the parafovea (see Veldre et al., 2020, for E-Z Reader simulations demonstrating that skipping effects can be attributed to postlexical integration). Indeed, even if predictability effects capture genuine prediction processes, the fact that some of these effects emerge on relatively late measures suggests that predictability effects may *also* have some postlexical impact. Given the key contributions of both prediction and integration processes, it is plausible that both are involved in the manifestation of predictability effects during reading (Brouwer et al., 2021; Ferreira & Chantavarin, 2018; Onnis et al., 2022).

#### Probabilistic inference processes

One final account of predictability effects is that they reflect a processing cost, specifically, the cost of *probabilistic inference*. This view derives from information theory (Hale, 2001; Levy, 2008), which, as described in section Sentence-processing models, posits that the primary role of the language processor is to assign a probability distribution over all possible continuations of a sentence. Under this account, processes such as prediction play no role in explaining predictability effects during reading because these effects, as indexed by surprisal, are simply a natural consequence of the probability distribution being updated with each upcoming word. In other words, in contrast to the view of sentence processing adopted by this article so far, which focuses on understanding how prediction contributes to building a mental representation of language structure and meaning (see Shain, 2024), the inferential view of sentence processing focuses only on the problem of how the language processor computes probabilistic inferences given limited evidence. Empirical support for this account comes from findings that, firstly, increasing surprisal has been linked to more effortful behavioral and neural processing (see section Surprisal and entropy); secondly, the relationship between predictability and processing effort appears to be logarithmic (see section What is the functional relationship between predictability and processing difficulty?); and thirdly, there is no specific cost when an unpredictable word is encountered in a context that predicts another completion (see section Graded preactivation processes; see Staub, 2024, for a review). At the same time, however, the findings that, firstly, predictability does not interact with frequency and, secondly, these two factors are dissociable across methodologies (see section Predictability and word frequency effects) remain a challenge to the inferential account because information theory hypothesizes that frequency effects function as predictability effects when context is absent (Hale, 2001; Levy, 2008; Norris, 2006). Thus, while probabilistic inference may provide an explanation for predictability effects to some extent, there appear to be limits on the scope of this account of cognitive processing. Indeed, this account does not deal with limitations in human attention and memory, which might lead to imperfect or “lossy” access to the linguistic context required by the language processor to make probabilistic inferences (Futrell et al., 2020; Hahn et al., 2022).

#### Future directions

Although the most intuitive explanation of predictability effects is that they reflect lexical prediction processes, this account cannot be definitively distinguished from processes of graded preactivation, integration, or probabilistic inference, which also predict processing benefits for predictable words in context. Indeed, as suggested throughout this section, it is likely that predictability effects reflect some combination of these processes. It will thus be important for ongoing research to clarify the processes that underlie predictability effects during reading. For example, the link between lexical prediction and graded preactivation processes could be strengthened by examining the factors and time course that underpin how readers transition from the partial preactivation to the specific prediction of upcoming words. These prediction-based processes may also be disentangled from general integration processes by using computational estimates generated before a target word is reached, which must reflect prediction processes, compared with when a target word is reached, which is more likely to capture some aspect of integration processes. Finally, it will also be a goal for future research to reconcile the underlying assumptions of differing views of sentence processing that have been used to explain predictability effects in order to provide a more integrated theory of language comprehension.

## Summary, conclusions, and future directions

This article provides an overview of the current literature relating to prediction processes during real-time language comprehension, with a focus on evidence from predictability effects during reading. The overall picture that emerges based on eye-tracking and brain-imaging research to date is that, to some extent, effects of predictability can be taken as demonstrating evidence that the language processor engages in genuine linguistic prediction during online processing. In particular, the investigation of several prominent theoretical issues lends support to the emerging view that prediction does not always entail the activation of a specific lexical item, but rather graded preactivation of multiple sources of information. Moreover, predictability effects do not solely reflect integration processes. Rather, they appear to operate at a very specific early stage of processing, possibly even before a word has been fixated. However, there is also accumulating evidence to suggest that factors like individual differences among readers, age, and different task demands can modulate readers’ use of prediction to varying degrees. Taken together then, readers’ ability to anticipate upcoming information does play a helping role in online processing; however, evidence that, in its strongest form, prediction depends on the availability of resources and time suggests that it may not always be absolutely necessary for successful language comprehension.

As mentioned throughout this article, however, there remain several unresolved theoretical issues which are important for understanding how prediction allows real-time language comprehension to unfold as rapidly and effortlessly as it does. The most important questions are summarized below:

1. Do computational estimates of predictability provide a viable alternative to cloze probability estimates in capturing the predictions of human comprehenders?
2. Is there compelling evidence that the effects of predictability arise during early stages of processing or even before the critical word has been encountered which would provide the clearest demonstration of prediction?
3. Is there evidence from factors including, but not limited to, individual differences, age, and task demands that prediction is a flexible strategy that depends on the availability of resources and time?
4. How can the processes underlying predictability effects be understood within theories of language comprehension that incorporate inferential views of sentence processing?
5. How can computer models of reading be used to account for how predictability facilitates the reading of text at both behavioral and neural levels?

Answering these questions will be key to progressing the literature from investigating whether prediction is an important component of real-time language comprehension towards understanding the cognitive architecture and mechanisms that support these computations.

One approach, in particular, that will facilitate future research into prediction processes during real-time language comprehension is the ongoing advancement of underutilized methodologies such as the co-registration of eye-movement and brain activity data. The recording of continuous brain activity under natural reading conditions affords a more comprehensive assessment of the behavioral and neural correlates underpinning online cognitive processing. Although these techniques face significant methodological and analytical challenges (see Degno et al., 2021; Himmelstoss et al., 2020, for reviews), they have the potential to contribute to resolving the issues outlined above and to reconcile the apparent discrepancies in existing conclusions of eye-tracking and brain-imaging research. Co-registration methods will also play a role in the development of computer models of reading as they attempt to simulate the time course of lexical and contextual factors on both behavioral and neural data with minimal additional assumptions.

To conclude, linguistic prediction during online processing is consistent with the growing realization that the brain constantly functions as a “prediction machine,” as posited by general predictive accounts of cognitive functioning (Clark, 2013; Friston, 2010). Although some aspects of how these processes unfold are still being investigated, debated, and refined, it is increasingly apparent that anticipatory prediction should no longer be considered a peripheral question in the literature but rather understood as a natural part of the way language unfolds.

## Data Availability

Not applicable.

## Footnotes

1 Although this article is about the role of prediction in reading, what has been learned about this topic will likely have broader implications for the more general understanding of language comprehension.

2 The values of these parameters (and those of the other models that will be discussed below) are typically selected to minimize the discrepancy between observed measures of reading behavior (e.g., mean fixation durations and probability measures) and the model’s predicted values of those same measures. It is also important to note that the explanation provided here ignores the additional complexity of retinal eccentricity; in E-Z Reader, *t*(*L*_1_) also increases as a function of the distance between the location of word *n* and the centre of vision due to decreases in visual acuity and increasing effects of crowding (see Veldre et al., 2023).

3 The MAZE task (Forster et al., 2009; Freedman & Forster, 1985) is another measure of online sentence processing in which two words are presented at the same time for each upcoming position of a sentence and participants must choose the word that grammatically continues the sentence.

4 Although participants are usually unaware of display changes, there is some evidence that display change awareness modulates preview effects, albeit inconsistently (cf. Veldre & Andrews, 2018a; White et al., 2005a). To date, however, there is no evidence that display change awareness affects the relationship between predictability effects and parafoveal processing.
